# Identification of Glutaminyl Cyclase Genes Involved in Pyroglutamate Modification of Fungal Lignocellulolytic Enzymes

**DOI:** 10.1128/mBio.02231-16

**Published:** 2017-01-17

**Authors:** Vincent W. Wu, Craig M. Dana, Anthony T. Iavarone, Douglas S. Clark, N. Louise Glass

**Affiliations:** a Department of Plant and Microbial Biology, University of California, Berkeley, Berkeley, California, USA; b Energy Biosciences Institute, University of California, Berkeley, Berkeley, California, USA; c Chemical and Biomolecular Engineering, University of California, Berkeley, Berkeley, California, USA; d QB3/Chemistry Mass Spectrometry Facility, University of California, Berkeley, Berkeley, California, USA; e Lawrence Berkeley National Laboratory, Berkeley, California, USA; Cornell University

## Abstract

The breakdown of plant biomass to simple sugars is essential for the production of second-generation biofuels and high-value bioproducts. Currently, enzymes produced from filamentous fungi are used for deconstructing plant cell wall polysaccharides into fermentable sugars for biorefinery applications. A post-translational N-terminal pyroglutamate modification observed in some of these enzymes occurs when N-terminal glutamine or glutamate is cyclized to form a five-membered ring. This modification has been shown to confer resistance to thermal denaturation for CBH-1 and EG-1 cellulases. In mammalian cells, the formation of pyroglutamate is catalyzed by glutaminyl cyclases. Using the model filamentous fungus *Neurospora crassa*, we identified two genes (*qc*-*1* and *qc*-*2*) that encode proteins homologous to mammalian glutaminyl cyclases. We show that *qc*-*1* and *qc*-*2* are essential for catalyzing the formation of an N-terminal pyroglutamate on CBH-1 and GH5-1. CBH-1 and GH5-1 produced in a *Δqc*-*1 Δqc*-*2* mutant, and thus lacking the N-terminal pyroglutamate modification, showed greater sensitivity to thermal denaturation, and for GH5-1, susceptibility to proteolytic cleavage. QC-1 and QC-2 are endoplasmic reticulum (ER)-localized proteins. The pyroglutamate modification is predicted to occur in a number of additional fungal proteins that have diverse functions. The identification of glutaminyl cyclases in fungi may have implications for production of lignocellulolytic enzymes, heterologous expression, and biotechnological applications revolving around protein stability.

## IMPORTANCE

Pyroglutamate modification is the post-translational conversion of N-terminal glutamine or glutamate into a cyclized amino acid derivative. This modification is well studied in animal systems but poorly explored in fungal systems. In *Neurospora crassa*, we show that this modification takes place in the ER and is catalyzed by two well-conserved enzymes, ubiquitously conserved throughout the fungal kingdom. We demonstrate that the modification is important for the structural stability and aminopeptidase resistance of CBH-1 and GH5-1, two important cellulase enzymes utilized in industrial plant cell wall deconstruction. Many additional fungal proteins predicted in the genome of *N. crassa* and other filamentous fungi are predicted to carry an N-terminal pyroglutamate modification. Pyroglutamate addition may also be a useful way to stabilize secreted proteins and peptides, which can be easily produced in fungal production systems.

## INTRODUCTION

**FIG 1:**
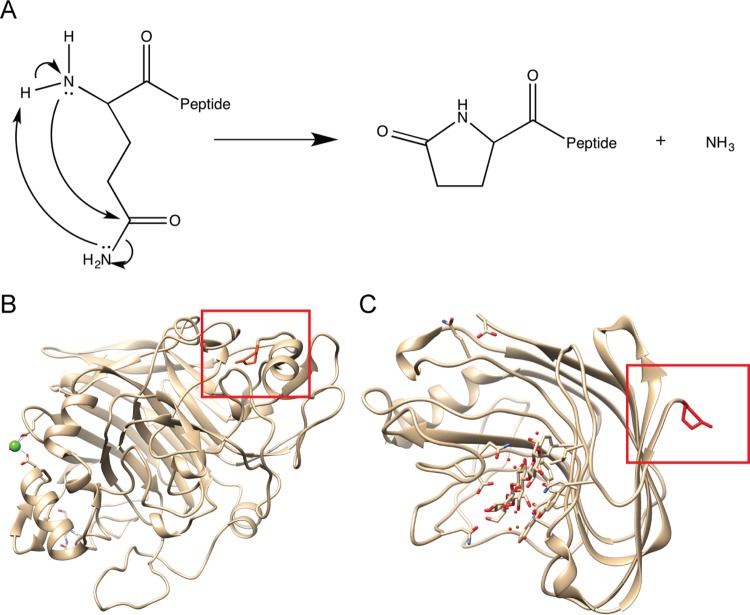
Pyroglutamate formation and its presence and function in fungal hydrolytic enzymes. (A) Mechanism of pyroglutamate formation from N-terminal glutamine. Conversion of glutamine to pGlu releases one ammonia molecule. (B) Crystal structure of CBH-1 (GH7 family) from *Trichoderma reesei* shows pGlu-modified N terminus with pGlu distinctly tucked into a pocket within the protein (56). (C) Crystal structure of Cel12A (GH12 family) from *Humicola grisea* provides an example of a pGlu-modified N terminus typical of non-GH7 family proteins (57). Crystal structure images generated with Chimera 1.10.2 (58) from structures fetched from the RCSB Protein Data Bank (PDB identifier [ID] or code 1CEL and 1UU4).

A barrier to the production of fuels and value-added chemicals from lignocellulose is the conversion of insoluble polysaccharides found in plant biomass to fermentable sugars (1). Current industrial processes utilize hydrolytic enzymes produced by filamentous fungi for depolymerization of plant cell wall polysaccharides into sugars that are subsequently used in fermentation and chemical conversion processes (2). Research on the characterization of lignocellulolytic enzymes in fungi is extensive, and crystallography studies have revealed important posttranslational modifications. For example, the crystal structure of cellobiohydrolase 1 (CBH-1), a cellobiohydrolase belonging to the glycosyl hydrolase 7 (GH7) family, revealed an N-terminal five-membered cyclic ring structure known as pyroglutamate (pGlu) (Fig. 1A). N-terminal pGlu modification has been reported in a wide variety of proteins found in plants, animals, and bacteria and is catalyzed by glutaminyl cyclases (QCs) (3–5). In mammalian systems, a secreted QC catalyzes the pGlu modification of amyloid peptides, which are associated with increasing the risk of Alzheimer’s disease (6). The importance of pGlu modification was first demonstrated for its role in function and stability of peptide hormones and signaling molecules (7–9), snake venom peptides, and certain RNases (10, 11). Less is known about the roles of functionally convergent QCs in plants, but pGlu modification has been detected in a small number of proteins involved in wounding and pathogen response (4).

Although pGlu modification is evident in crystal structures of a number of lignocellulolytic enzymes in filamentous fungi, the biological role of this modification is unclear. However, two recent studies showed that heterologous production of a CBH-1 and an endoglucanase 1 (EG1) from a thermotolerant filamentous fungus *Talaromyces emersonii* in *Saccharomyces cerevisiae* showed decreased thermal stability (12, 13) (Fig. 1B). The thermal stability of *T. emersonii* CBH-1 (TeCBH-1) and EG1 purified from *S. cerevisiae* was rescued following treatment with a human QC enzyme.

Here, we explore pGlu formation and function using the lignocellulolytic fungus *Neurospora crassa* as a model system and identify two functionally redundant endoplas mic reticulum (ER)-localized enzymes that are essential for pGlu modification of a GH7 enzyme (TeCBH-1 and native CBH-1) and a GH5 enzyme (endoglucanase GH5-1). We show that the pGlu modification of CBH-1 and GH5-1 enhances thermostability, and for GH5-1, it also enhances resistance to N-terminal proteolytic cleavage. The development of an *N. crassa* system devoid of pGlu modification will allow further investigations into the diverse roles of pGlu modifications on a variety of proteins that traffic through the secretory pathway in filamentous fungi and may enable the development of tools for biotechnological applications.

## RESULTS

### Predicted glutaminyl cyclase genes in fungal genomes.

**FIG 2:**
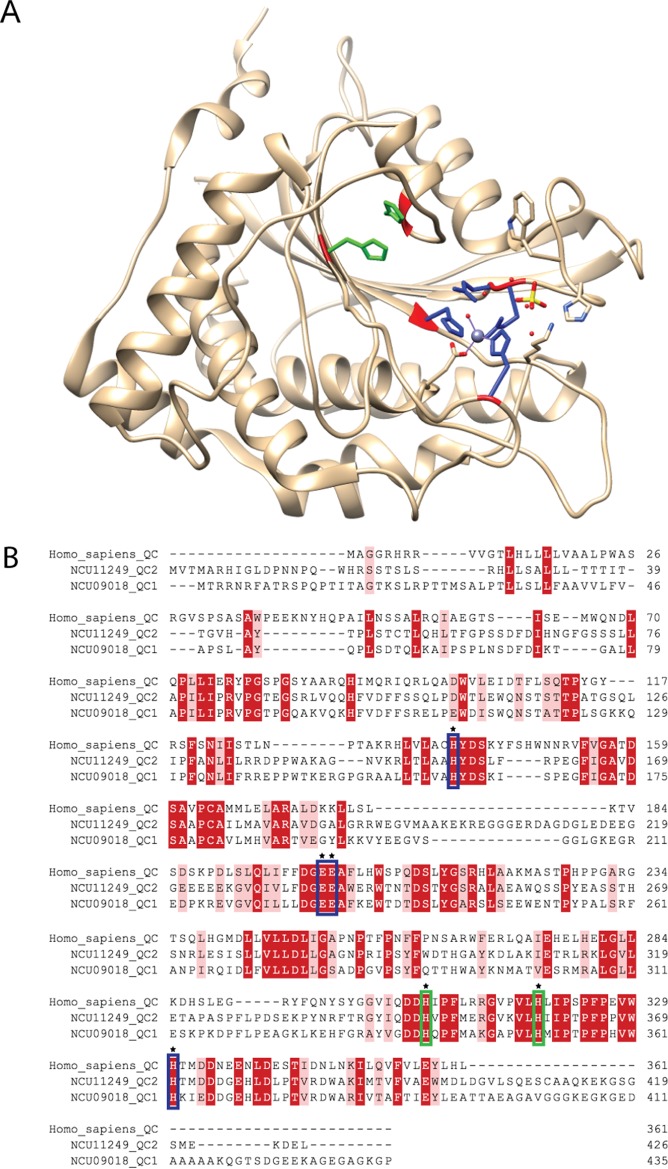
Conservation between *N*. *crassa* QC-1/QC-2 and human QC. (A) Crystal structure of human glutaminyl cyclase (59) showing functionally critical conserved residues with predicted glutaminyl cyclases from *N. crassa* (QC-1 and QC-2) (PDB ID 2AFM). Catalytic residues H140, E201, E202, and H330 are shown in blue. Substrate binding residues H309 and H317 are shown in green. (B) Multiple-sequence alignment of amino acid sequences from human QC and *N. crassa* QC-1 and QC-2. Identical amino acids (red), similar amino acids (pink), and absent amino acids (dashes) are indicated. Amino acids boxed in blue and green are the same catalytic and substrate binding residues as described above. Gaps introduced to maximize sequence alignment are indicated by dashes.

We hypothesized that fungi have uncharacterized QC enzymes that catalyze pGlu formation in lignocellulolytic enzymes. We searched the *N. crassa* genome for homologs to the human pituitary QC (*QPCT* GeneID 25797) biochemically shown to catalyze pGlu formation (14). We identified two genes, NCU09018 and NCU11249, that encode predicted proteins with significant similarity to human QC (~25% to 30% amino acid identity). The NCU09018 and NCU11249 proteins show conservation of four catalytic residues (H140, E201, E202, and H330) and two substrate binding residues (H309 and H317) that are important for function of human QC enzymes (Fig. 2) (14). We named NCU09018 and NCU11249 *qc*-*1* and *qc*-*2*, respectively.

Using *qc*-*1* and *qc*-*2* amino acid sequences as queries, we built a maximum likelihood phylogeny of fungal QC proteins (see Fig. S1 in the supplemental material). The genomes of the majority of ascomycete fungi have one predicted gene with high similarity to *N. crassa qc*-*1* and/or *qc*-*2* (*qc*-*1*/*qc*-*2*). Homologs to *qc*-*1*/*qc*-*2* were identified in other major lineages in fungi, including the Chytridiomycota, Zygomycota, Glomeromycota, and Basidiomycota. The phylogeny additionally showed that species with two QC genes is the derived state of a small subclade of species within the Sordariomycetes (Fig. S1).

10.1128/mBio.02231-16.1 FIG S1  QC phylogeny. Maximum likelihood tree built from homologs of *qc*-*1* identified in fungal genomes using animals as the outgroup. Bootstrap values generated from 100 replicates are labeled at each node. Tree branches in orange highlight species with secondary copy of QC and represent a subclade within Sordariomycetes that contains *N. crassa* and closely related species. Representatives from various classes within Ascomycota are highlighted in colored boxes and labeled in green. Download FIG S1, TIF file, 0.9 MB. Copyright © 2017 Wu et al. 2017 Wu et al. This content is distributed under the terms of the Creative Commons Attribution 4.0 International license.

### Deletion of the two predicted glutaminyl cyclase genes (*qc*-*1* and *qc*-*2*) in *N. crassa* results in thermal instability of CBH-1.

**FIG 3:**
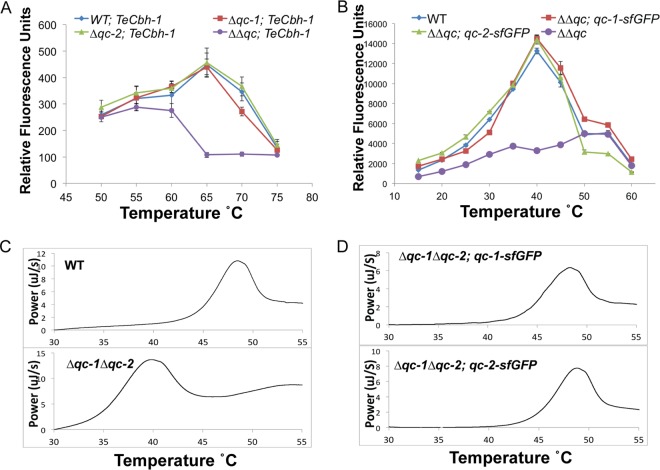
TeCBH-1/CBH-1 activity in the *Δqc*-*1 Δqc*-*2* strain and cellular localization of QC-1 and QC-2. (A) MuLac activity between 50°C and 75°C of supernatants from WT, *Δqc*-*1*, *Δqc*-*2*, and *Δqc*-*1 Δqc*-*2* strains expressing TeCBH-1. (B) MuLac activity between 15°C and 60°C of purified CBH-1 from WT or *Δqc*-*1 Δqc*-*2* cells or from the *Δqc*-*1 Δqc*-*2* mutant containing *qc*-*1*-*sfGFP* or *qc*-*2*-*sfGFP*. Error bars in panels A and B represent standard deviations from quadruplicate experiments. (C and D) Melting temperature of CBH-1 purified from WT or *Δqc*-*1 Δqc*-*2* cells as measured by differential scanning calorimetry.

To determine whether *qc*-*1* and/or *qc*-*2* are important for pGlu formation in CBH-1, we constructed strains carrying deletions of *qc*-*1* (*Δqc*-*1*), *qc*-*2* (*Δqc*-*2*), and both QCs (*Δqc*-*1* Δ*qc*-*2*). The *T. emersonii cbh*-*1* coding sequence regulated by the glyceraldehyde-3-phosphate dehydrogenase (GPD) promoter (13) was introduced into the wild type (WT) and the *Δqc*-*1*, *Δqc-2*, and *Δqc*-*1 Δqc*-*2* strains. Activity of TeCBH-1 culture supernatants was assayed from 35°C to 60°C using a 4-methylumbelliferyl β-d-lactopyranoside (MuLac) assay for enzyme activity (Fig. 3A) (see Materials and Methods). TeCBH-1 from WT cells and single *Δqc*-*1* or *Δqc*-*2* mutants displayed high activity (Fig. 3A). In contrast, TeCBH-1 from the Δ*qc*-*1* Δ*qc*-*2* mutant showed reduced activity at high temperatures, an effect especially noticeable between 60°C and 65°C (Fig. 3A). These results recapitulated previous data demonstrating a difference in activity in pGlu-modified versus unmodified TeCBH-1 at different temperatures (13). Furthermore, these data suggested that the *N. crassa* QC enzymes are functionally redundant.

To determine whether the role of *qc*-*1* and *qc*-*2* in the thermotolerance of heterologously expressed TeCBH-1 was unique, we investigated the thermotolerance of native CBH-1 (NCU07340) in WT cells versus the *Δqc*-*1 Δqc*-*2* mutant. Similar to the results with TeCBH-1, native CBH-1 purified from the Δ*qc*-*1* Δ*qc*-*2* mutant showed low enzymatic activity between 25°C and 40°C (Fig. 3B), while CBH-1 purified from WT cells showed an increase in enzyme activity between 25°C and 40°C. To confirm that the effect on thermostability of CBH-1 was dependent upon functional *qc*-*1* and *qc*-*2*, the *Δqc*-*1 Δqc*-*2* strain was transformed with either *qc*-*1* or *qc*-*2* constructs epitope tagged with in-frame superfolder green fluorescent protein (sfGFP) (*qc*-*1*-*sfGFP* or *qc*-*2*-*sfGFP*). The activity curve of CBH-1 purified from the *Δqc*-*1 Δqc*-*2* mutant bearing either *qc*-*1*-*sfGFP* or *qc*-*2*-*sfGFP* was similar to that of CBH-1 isolated from WT cells, indicating that QC function in *N. crassa* is redundant under these experimental conditions. Importantly, both QC-1 and QC-2 were essential for thermostability of TeCBH-1 and CBH-1 in *N. crassa*.

To further define the difference in thermal stability of native CBH-1, we performed differential scanning calorimetry to determine the melting temperatures (*T*_*m*_) of purified CBH-1 from WT cells versus CBH-1 purified from *Δqc*-*1 Δqc*-*2* mutant cells. CBH-1 purified from WT cells showed a melt peak at ~48°C, while CBH-1 purified from the *Δqc*-*1* Δ*qc*-*2* cells showed a melt peak at ~39°C (Fig. 3C). Consistent with previous results, the melting curves of CBH-1 purified from the *Δqc*-*1 Δqc*-*2* mutant bearing either *qc*-*1*-*sfGFP* or *qc*-*2*-*sfGFP* were very similar to that of CBH-1 purified from WT cells (Fig. 3D).

### Mass spectrometry of CBH-1 purified from WT cells shows the presence of an N-terminal pyroglutamate modification.

**FIG 4:**
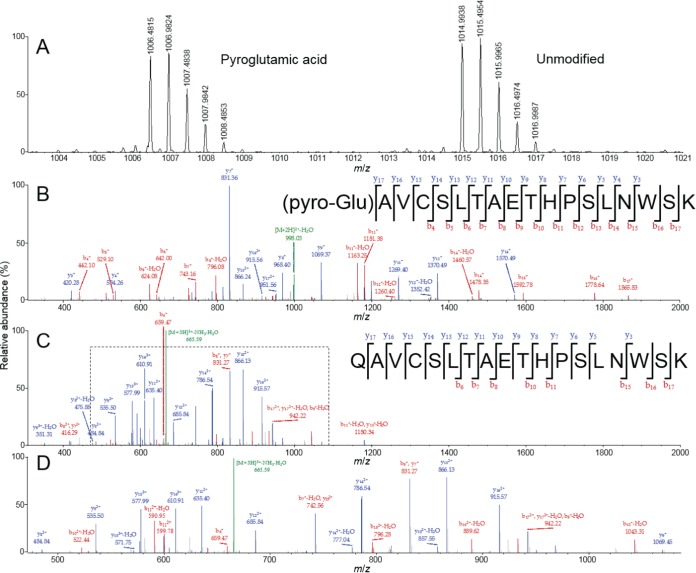
LC-MS/MS of *N. crassa* CBH-1. (A) Full-scan mass spectrum showing detail for isotopically resolved, doubly charged, positive precursor ions occurring at monoisotopic mass-to-charge ratios (*m*/*z*) of 1,006.4815 and 1,014.9938, due to the [M+2H]^2+^ ions of the pGlu-modified and unmodified QAVCSLTAETHPSLNWSK peptide. (B and C) Annotated MS/MS spectra and peptide sequence maps resulting from collision-induced dissociation (CID) of precursor peptide ions containing pGlu-modified N terminus (precursor ion *m*/*z* = 1,006.4815, [M+2H]^2+^ ion) (B) and unmodified N terminus (*m*/*z* = 676.9987, [M+3H]^3+^ ion) (C). (D) Detail for the region denoted by the dashed line in panel C. MS/MS spectra are annotated using the nomenclature of Roepstorff and Fohlman (55).

Previous studies describing pGlu function in CBH-1 did not verify the presence or absence of the N-terminal pGlu modification (12, 13). To address this question directly, we conducted liquid chromatography-tandem mass spectrometry (LC-MS/MS) analysis of trypsin-digested CBH-1 purified from WT cells versus that from the Δ*qc*-*1* Δ*qc*-*2* mutant; the N-terminal CBH-1 peptide resulting from trypsin digestion is QAVCSLTAETHPSLNWSK. Formation of pyroglutamate from an N-terminal glutamine residue results in a decrease in exact (monoisotopic) molecular mass of 17.0265 Da, due to elimination of a molecule of ammonia. The difference in molecular mass between N-terminal peptides with and without pyroglutamate was discerned in precursor ions measured in full-scan mass spectra (Fig. 4A). MS/MS spectra pinpointed the site of pyroglutamate formation as the N terminus (Fig. 4B to D). For CBH-1 purified from WT cells, only the pGlu-modified peptide was detected. For CBH-1 purified from the *Δqc*-*1 Δqc*-*2* mutant, both unmodified N-terminal glutamine and pGlu were detected at an abundance ratio of 9:1, respectively (Table S1). The small fraction of pGlu-modified CBH-1 in the Δ*qc*-*1* Δ*qc*-*2* mutant may be due to spontaneous cyclization of N-terminal glutamine, a phenomenon previously observed for mammalian proteins (7). Altogether, these data strongly support the hypothesis that QC-1 and QC-2 are essential for the N-terminal pGlu modification of CBH-1 in *N. crassa*.

10.1128/mBio.02231-16.3 TABLE S1  Peptide sequences and modifications detected by LC-MS/MS of trypsin-digested CBH-1 Download TABLE S1, DOCX file, 0.1 MB. Copyright © 2017 Wu et al. 2017 Wu et al. This content is distributed under the terms of the Creative Commons Attribution 4.0 International license.

### pGlu formation occurs in the endoplasmic reticulum.

**FIG 5:**
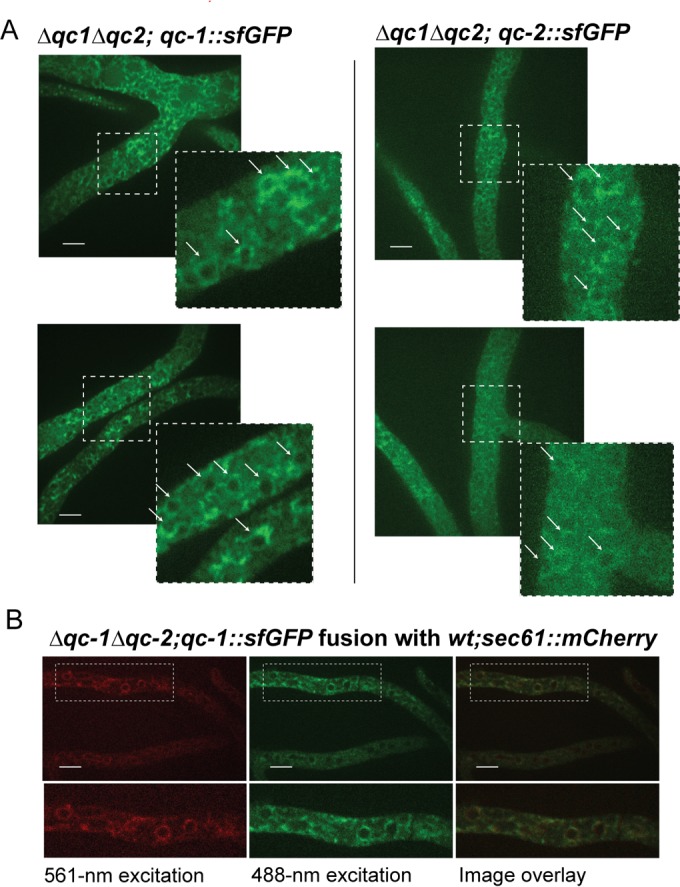
Intracellular localization of QC-1 and QC-2. (A) Fluorescence microscopy of *Δqc*-*1 Δqc*-*2* strain expressing either *qc*-*1*-*sfGFP* or *qc*-*2*-*sfGFP*. (B) Fluorescence microscopy of heterokaryotic cells expressing *qc*-*1*-*sfGFP* and *sec61*-*mCherry* (53). Bars, 10 μm.

In mammalian cells, two QC enzymes catalyze pGlu formation. One QC enzyme has an N-terminal membrane anchor and localizes to the Golgi compartment; direct targets of this QC are unknown (15). The second QC is cosecreted with its protein substrates (16); the secreted QC is likely responsible for cyclizing prohormones that mature in secretory granules. We reasoned that *N. crassa* QC-2 resided in the endoplasmic reticulum (ER) due to the presence of a signal peptide and a KDEL ER retention sequence (17) (Fig. 2B). QC-1 also has a signal peptide but no KDEL sequence. The *Δqc*-*1 Δqc*-*2* strains bearing either *qc*-*1*-*sfGFP* or *qc*-*2*-*sfGFP* (that fully complemented the CBH-1 phenotype of the *Δqc*-*1 Δqc*-*2* mutant; Fig. 3B) were subjected to fluorescence microscopy. QC-1–sfGFP and QC-2–sfGFP fluorescence was observed in structures inside the cell that primarily ringed nuclei, a localization pattern consistent with the ER of filamentous fungi (Fig. 5A) (18). Differences in the cellular localization of QC-1 or QC-2 in hyphae were not apparent, although strains carrying QC-2–sfGFP had lower GFP signal. To further confirm ER localization of QC-1, colocalization with the ER resident protein Sec61 (19) was performed. A heterokaryotic strain expressing Sec61-mCherry and QC-1–sfGFP showed an identical intracellular localization (Fig. 5B), indicating that QC-1 localizes to the ER.

### pGlu prevents N-terminal truncation of *N. crassa* GH5-1.

The crystal structure of CBH-1 from a number of filamentous fungi shows an N-terminal pGlu modification (20, 21). The crystal structures of additional GH7 members, including EG1 (Cel7b) (22, 23) and Cel7D (24) have an N-terminal pGlu. A pGlu modification is also observed in the crystal structure of fungal hydrolases outside the GH7 family, including Cel12A, cellobiose dehydrogenase (Cdh1) and GH10 xylanases (25–28). However, unlike GH7 family enzymes, these enzymes display a pGlu that does not fit within a hydrophobic pocket but rather lies in an extended tail at a distance to the catalytic and substrate binding sites of the protein. This structural aspect of the pGlu modification of *Trichoderma reesei* Cel12A is shown in Fig. 1C, in contrast to GH7 enzymes like CBH-1 (Fig. 1B).

**FIG 6:**
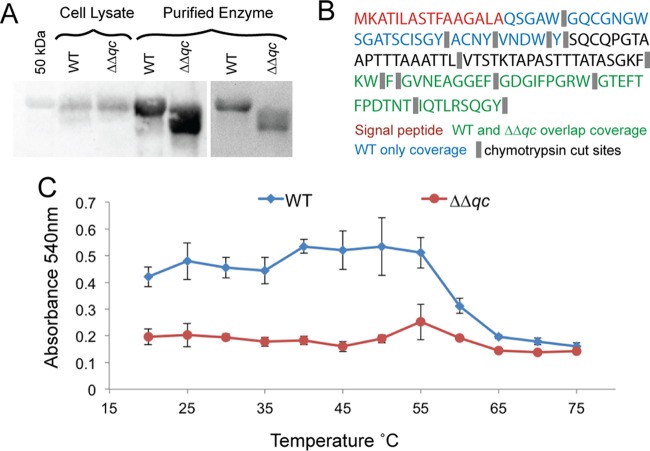
Role of pGlu in GH5-1. (A) Western blotting and Coomassie blue staining of GH5-1. (Right) Coomassie blue staining of GH5-1 purified from WT and *Δqc*-*1 Δqc*-*2* cells expressing GH5-1-6xHis. (Left) Western blotting using anti-GH5-1 antibody on cell lysates versus purified GH5-1 from the same strains. (B) Peptide detection by LC-MS/MS of chymotrypsin-digested GH5-1-6xHis expressed in WT versus *Δqc*-*1* Δ*qc*-*2* cells. Only the first 135 amino acids of the GH5-1 sequence are shown. (C) Endoglucanase activity of purified GH5-1 from WT versus *Δqc*-*1 Δqc*-*2* cells from 20°C to 75°C as measured by a filter paper assay (54). Error bars represent standard deviations from quadruplicate experiments.

We therefore investigated endoglucanase II (*gh5*-*1*; NCU00762), a non-GH family 7 protein that is a highly expressed cellulase in *N. crassa* and has been previously characterized (29). This enzyme has a clear predicted N-terminal glutamine after the signal peptide. To determine whether GH5-1 contained an N-terminal pGlu modification, we constructed strains expressing 6×His-tagged GH5-1 under the GPD promoter and purified GH5-1 from WT and *Δqc*-*1 Δqc*-*2* cells. Purified GH5-1 was subsequently subjected to chymotrypsin digestion and LC-MS/MS analysis. As with CBH-1, pGlu-modified GH5-1 was detected only in the WT sample, whereas neither a pGlu-modified nor unmodified GH5-1 N-terminal peptide was detected in the *Δqc*-*1 Δqc*-*2* sample (Table S2). Further examination showed that the first 33 amino acids from the N terminus of GH5-1 were absent in the *Δqc*-*1* Δ*qc*-*2* sample (Fig. 6B; Table S2).

10.1128/mBio.02231-16.4 TABLE S2  GH5-1 peptides detected by LC-MS/MS from GH5-1 digested with chymotrypsin Download TABLE S2, DOCX file, 0.1 MB. Copyright © 2017 Wu et al. 2017 Wu et al. This content is distributed under the terms of the Creative Commons Attribution 4.0 International license.

We reasoned that if GH5-1 from *Δqc*-*1 Δqc*-*2* cells was N-terminally truncated, it should have a reduced molecular weight compared to GH5-1 from WT cells. In support of our hypothesis, GH5-1 purified from *Δqc*-*1 Δqc*-*2* cells showed a band at ~47 kDa, a lower molecular mass than GH5-1 purified from WT cells (~50 kDa) (Fig. 6A). Furthermore, GH5-1 from *Δqc*-*1 Δqc*-*2* cells ran as a wider-than-expected protein band compared to GH5-1 from WT cells, suggesting that GH5-1 from *Δqc*-*1 Δqc*-*2* cells may be subject to truncations at various N-terminal locations.

We hypothesized that GH5-1 could be truncated either extracellularly or by proteolytic processing during passage through the secretory system. To differentiate these possibilities, we subjected whole-cell lysates from WT and *Δqc*-*1 Δqc*-*2* cells to immunoprecipitation and Western blotting with anti-GH5-1 antibody. As shown in Fig. 6A, no difference in molecular mass was observed between intracellular GH5-1 from WT cells versus the *Δqc*-*1 Δqc*-*2* cells, indicating that the loss of the N-terminal region of GH5-1 from *Δqc*-*1 Δqc*-*2* cells occurred after secretion. *N. crassa* has a number of extracellular proteases and peptidases (30), which we hypothesize are responsible for the truncation of unmodified GH5-1.

To test whether truncation of GH5-1 has consequences on enzymatic activity, we subjected purified GH5-1 to a modified filter paper assay. Purified GH5-1 from WT and *Δqc*-*1 Δqc*-*2* cells was incubated with 1-cm squares of Whatman cellulose filter paper across a range of temperatures and released sugar reducing ends were reacted with dinitrosalicylic acid (DNS). GH5-1 from *Δqc*-*1 Δqc*-*2* cells showed reduced enzymatic activity between 20°C and 55°C compared to GH5-1 purified from WT cells (Fig. 6C). At temperatures above 55°C, both enzymes are inactive and show only low levels of endoglucanase activity (Fig. 6C).

### Proteins with a predicted pGlu modification.

Human QC cyclizes exposed N-terminal glutamine (Q) or glutamate (E) residues (31). We hypothesized that *N. crassa* proteins with an exposed N-terminal Q or E following signal peptide cleavage would be modified to pGlu when trafficked through the ER. Thus, we sorted predicted proteins destined for the secretory pathway with an N-terminal Q or E (Table S3); 90 proteins were identified. As expected, CBH-1, GH5-1, CDH-1, and GH7-2 (EG1) were included in this list. Twelve additional plant cell wall-degrading enzymes were identified; many of these enzymes have been detected in the secretome of *N. crassa* (32–34). The two largest remaining groups are proteins involved in modulation of the fungal cell wall (35–37) and secreted proteases. The remaining proteins have a range of functions, including oxidoreductases, nucleotidases, phosphatases, the ER resident protein IRE-1 and several vacuolar proteins. Finally, there are 47 proteins with no known functions, 19 of which show conservation among ascomycete fungi outside the Sordariomycetes.

10.1128/mBio.02231-16.5 TABLE S3  Predicted *N. crassa* pGlu-modified proteins Download TABLE S3, DOCX file, 0.1 MB. Copyright © 2017 Wu et al. 2017 Wu et al. This content is distributed under the terms of the Creative Commons Attribution 4.0 International license.

## DISCUSSION

Secreted glutaminyl cyclases in mammals play a role in stability, function, and aggregation of secreted peptides and proteins (6, 7, 10, 11). pGlu modification is required for certain peptides for resistance to peptidases and to remain receptor active (7, 38). Here, we demonstrated that pGlu modification of secreted cellulase enzymes in *N. crassa* is catalyzed by two QCs that reside in the ER. Deletion of *N. crassa qc*-*1* and *qc*-*2* resulted in a significant reduction in pGlu-modified CBH-1 and GH5-1. In addition, CBH-1 and GH5-1 lacking the pGlu modification showed a reduction in enzyme activity, and unmodified GH5-1 was subject to N-terminal proteolytic degradation. These data support a role of pGlu in protecting the N termini of secreted proteins from degradation by endogenous and exogenous exopeptidases.

A second QC in mammals is retained within the Golgi complex (isoQC) (15, 39). Differential cellular distribution of QC and isoQC may indicate a preference for different substrates in the secretory pathway and thus distinct physiological roles. This has been demonstrated in monocyte chemoattractant proteins (MCPs) versus thyrotropin-releasing hormone (TRH) where only one QC is involved in the pGlu formation for each protein (39, 40). In *N. crassa*, both QC-1 and QC-2 localized primarily to the ER, where proteins with a liberated N-terminal Q or E would be cyclized to pGlu. It is unclear whether QC-1 or QC-2 may preferentially catalyze pGlu on particular proteins. Future studies on elucidating further and potentially specific targets of these two QC proteins in *N. crassa* will be informative.

TeCBH-1 and TeEG1 heterologously expressed in *S. cerevisiae* show reduced thermal stability and presumably lack the N-terminal pGlu modification (12, 13). In hindsight, this result is surprising because a QC homolog is present in the *S. cerevisiae* genome (which is uncharacterized, but a GFP-tagged version is reported to localize to the ER; YFR018C) (41) (see Fig. S1 in the supplemental material). However, to enhance expression in *S. cerevisiae*, an engineered alpha factor APPS4 prepro leader sequence (42) was appended to the N terminus of the *TeCBH*-*1* and *TeEG1* constructs. The leader sequence APPS4 contains a secondary KEX2 site after the signal peptide cleavage site, causing the N-terminal Q to be revealed at the Golgi compartment only where KEX2 cleavage occurs. If *S. cerevisiae* QC is ER localized like *N. crassa* QCs, pGlu formation would not occur when using APPS4 as a leader sequence, resulting in TeCBH-1 and TeEG1 proteins that lack this modification and thus are thermally less stable.

Fungal systems may prove useful for the study and production of pGlu-modified proteins. Recent studies have indicated that pGlu-modified protein production is a difficult process, where complex expression systems or postexpression treatment with purified QC is required (43, 44). As shown in this study, simple procedures can be used to tag, express, and purify secreted proteins from the WT and from Δ*qc*-*1* Δ*qc*-*2* strains. Compared to other expression systems, filamentous fungi have the advantage of being eukaryotic, with high protein production strains (45). Appending pGlu to nonmodified proteins may be a useful tool for heterologous expression of proteins in filamentous fungi for biotechnological applications. Where the N termini are not involved in essential functions, pGlu addition could maintain protein stability especially in systems where exopeptidases are ubiquitous.

We identified ~90 proteins from *N. crassa* predicted to be pGlu modified. The majority of these proteins are predicted to be secreted and include plant cell wall-degrading enzymes, proteases, fungal cell wall proteins, and putative small secreted peptides of unknown function. Plant-pathogenic fungi secrete a diverse set of small, secreted effector proteins crucial for pathogenesis (46). Since many pathogenic fungi also have genes encoding QCs in their genome (Fig. S1), it is possible that the pGlu plays a role in the function/stability of these effector proteins. We also identified a few intracellular proteins with a predicted pGlu modification, including the ER protein Ire1p, which is important for triggering the unfolded protein response (47). The predicted pGlu site is well conserved among *IRE1* homologs in filamentous ascomycete fungi, but it is not conserved in *S. cerevisiae* Ire1p. The N-terminal domain of Ire1p has been shown to be important for binding to hydrophobic regions of unfolded proteins (47), and pGlu hydrophobicity in IRE-1 in filamentous fungi could potentially play a role in this process. Future experiments to dissect the role of pGlu modification in secreted and intracellular proteins will address these questions.

## MATERIALS AND METHODS

### Phylogeny.

Protein sequences were obtained by BLAST using NCU09018 (QC-1) and NCU11249 (QC-2) as protein search queries. Sequences were aligned using MAFFT version 7 (48). The alignment was used to construct a maximum likelihood phylogeny using RAxML (49). FigTree v1.4.2 (http://tree.bio.ed.ac.uk/software/figtree/) was used for visualization.

### Strain construction.

Cassettes consisting of a hygromycin B resistance marker flanked by 3′ and 5′ regions of NCU11249 (*qc*-*2*) or NCU09018 (*qc*-*1*) were obtained from the Dunlap lab (Geisel School of Medicine, Dartmouth, NH). Cassettes were transformed into a Δ*mus*-*52* strain as described previously (50). Hygromycin B-resistant strains were verified to have the QC deletion through PCR analysis (see Fig. S2 in the supplemental material). Strains were crossed back to *N. crassa* FGSC 2489 and *qc*-*1* or *qc*-*2* deletion strains lacking Δ*mus*-*52* were identified; strains are available at the Fungal Genetics Stock Center (FGSC). The Δ*qc*-*1* Δ*qc*-*2* strain was generated by crossing Δ*qc*-*1* and Δ*qc*-*2* strains and screening ascospore progeny for the *qc*-*1* and *qc*-*2* deletions by PCR (Fig. S2C). For complementation, *qc*-*1* and *qc*-*2* were amplified from genomic DNA with additional restriction sites and fused to super folder GFP (51). The forward primer GAAAGGATCCATGACACGACGCAACCGCTT and reverse primer GATTAATTAAAGGCCCTTTCCCAGCA for *qc*-*1* and the forward primer GAAAGGATCCATGGTGACGATGGCACG and reverse primer GATTAATTAAGAGCTCGTCTTTCTCCATGCTC for *qc*-*2* included restriction sites for BamHI and PacI for cloning. Each insert was cloned into the pCSR1::GPD vector (13, 52), transformed into a Δ*qc*-*1* Δ*qc*-*2* strain and selected for cyclosporine resistance as described previously.

10.1128/mBio.02231-16.2 FIG S2  Genotype PCRs for *Δqc*-*1*, *Δqc*-*2*, and *Δqc*-*1 Δqc*-*2*. (A) PCR on genomic DNA amplified from seven homokaryotic strains using the forward primer ATGCGCAACCAATCAGAAGG from *qc*-*1* 5′ flanking region and reverse primer from the hygromycin resistance marker GACCGATGGCTGTGTAGAAGT to verify integration of hygromycin resistance into the *qc*-*1* locus and deletion of the *qc*-*1* ORF. (B) PCR on genomic DNA isolated from seven homokaryotic strains using forward primer GGCTGTTAGGGTAGAGCACA from *qc*-*2* 5′ flanking region and reverse primer from the hygromycin resistance marker GACCGATGGCTGTGTAGAAGT to verify integration of hygromycin resistance into the *qc*-*2* locus and deletion of the *qc*-*2* ORF. (C) Same PCRs as described above conducted on three strains resulting from a cross between *Δqc*-*1* and *Δqc*-*2* strains demonstrating progeny that carry deletions of both genes. WT FGSC 2489 genomic DNA was used as a negative control for these PCRs. Download FIG S2, TIF file, 0.3 MB. Copyright © 2017 Wu et al. 2017 Wu et al. This content is distributed under the terms of the Creative Commons Attribution 4.0 International license.

The GH5-1 open reading frame (ORF) (NCU00762) was PCR amplified from *N. crassa* genomic DNA with an additional 6×His sequence and restriction sites and inserted into the pCSR1::GPD vector (13). The forward primer AATCAAAGCGGCCGCATGAAGGCTACGATTCTTGCCA and reverse primer AAATTAATTAATTAATGATGATGATGATGATGAGGGGTATAGGTCTTGAGAAGG included restriction sites for NotI and PacI restriction enzymes. The TeCBH-1 in pCSR1::GPD vector (13) and GH5-1 in the pCSR1::GPD were transformed into *N. crassa* as described previously (52). Homokaryotic transformants were obtained as described above. The Sec61::mCherry strain was described previously (53).

### Fluorescence microscopy.

Confocal microscopy was performed as described previously (53) using a Leica SD6000 microscope with a 100× 1.4-numerical-aperture (NA) oil immersion objective equipped with a Yokogawa CSU-X1 spinning disc head and a 488-nm or 561-nm laser controlled by MetaMorph software. ImageJ software was used for false color and overlaying images.

### CBH-1 and GH5-1 purification.

*N. crassa* CBH-1 was purified from WT, *Δqc*-*1 Δqc*-*2*, and *Δqc*-*1 Δqc*-*2* complemented with either *qc*-*1*-*sfGFP* or *qc*-*2*-*sfGFP*. GH5-1 was purified from WT and *Δqc*-*1 Δqc*-2 strains expressing 6×His-tagged GH5-1 in the *csr*-*1* locus under the GPD promoter. Conidia from these homokaryotic strains were harvested and used to inoculate 1 liter of Vogel’s minimal medium (VMM) in a 2-liter flask at a concentration of 10^6^ conidia/ml.

For CBH-1 cultures, 2% (wt/vol) Avicel (Sigma-Aldrich) was added to the cultures after 2 days of growth to induce CBH-1 production and grown for an additional 3 days before harvesting. GH5-1 cultures were grown for a total of 3 days in VMM. All cultures were grown at 25°C and 200 rpm.

The cultures were filtered through a glass fiber filter and Corning 22-μm polyethersulfone (PES) bottle top filter. The supernatant was concentrated 10-fold using a Pellicon 5-kDa filter cassette, and proteins were precipitated using 45 g ammonium sulfate. Proteins were pelleted and resuspended in 20 mM Tris-HCl (pH 8.5) and desalted using HiPrep 26/10 desalting column (GE Healthcare Life Sciences). CBH-1 was separated from other extracellular proteins using the anion exchange column MonoQ 10/100 (GE Healthcare Life Sciences), while a GE Healthcare Life Sciences 1-ml His-Trap HP column (GE Healthcare Life Sciences) was used in tandem with MonoQ 10/100 column to separate GH5-1 from other extracellular proteins.

### MuLac assay for CBH-1.

Purified *N. crassa* CBH-1 enzyme at 15 µg/ml and MuLac (4-methylumbelliferyl β-d-lactopyranoside; Sigma) at a final concentration of 1 mM were incubated together in 0.05 M sodium acetate buffer (pH 5) in 50-µl volumes in VWR 12-well 0.2-ml PCR strip tubes. Strip tubes were incubated for 15 min across temperatures using Applied Biosystems Veriti 96-well thermocycler in veriflex mode. After 15 min, the temperature was raised to 95°C for 5 min to deactivate the enzyme. Reactants were transferred to Corning half-area-well 96-well clear bottom plates, and fluorescence was measured at 445 nm after excitation with 365 nm.

To assay TeCBH-1 from culture supernatant, MuLac (final concentration of 1 mM) was incubated with 25 µl of filtered culture supernatant for a final 50-µl volume in 0.05 M sodium acetate buffer (pH 5). Strip tubes were incubated using Applied Biosystems Veriti 96-well thermocycler in veriflex mode for 1 h and raised to 95°C for 5 min to deactivate the enzyme. The 445-nm emission/365-nm excitation was measured after incubation.

### Filter paper assay.

Purified GH5-1 was assayed using a modified filter paper assay (54). Folded 1-cm squares of Whatman no. 1 filter paper were incubated with purified GH5-1 in 0.05 M citrate buffer (pH 4.8) at a concentration of 7.5 µg/ml. The strips were placed in tubes and incubated in an Applied Biosystems Veriti 96-well thermocycler in veriflex mode holding every two wells at different temperatures and testing six temperatures per 12-well strip tube. Wells were held at temperatures between 20 and 45°C or 50 to 75°C at 5°C increments for 24 h. After incubation, 100 µl of 3,5-dinitrosalicylic acid reagent was added to assay for oligosaccharide reducing ends. Samples were diluted and measured for absorbance at 540 nm.

### Differential scanning calorimetry.

Purified CBH-1 at ~500 ng/µl in a 20 mM Tris-HCl (pH 8.5) buffer was loaded into the sample chamber Nano DSC (TA Instruments) and assessed for stability by differential scanning calorimetry according to reference 13.

### Liquid chromatography-mass spectrometry.

Trypsin-digested protein samples were analyzed using a Thermo-Dionex UltiMate3000 RSLCnano liquid chromatograph that was connected in-line with an LTQ-Orbitrap-XL mass spectrometer equipped with a nanoelectrospray ionization (nanoESI) source (Thermo Fisher Scientific, Waltham, MA). The LC was equipped with a C_18_ analytical column (Acclaim PepMap 100; Thermo) (150-mm length, 0.075-mm inner diameter, 3-µm particles, 100-Å pores) and a 1-µl sample loop. Acetonitrile (Fisher Optima grade, 99.9%), formic acid (1-ml ampules, 99+%; Thermo Pierce), and water purified to a resistivity of 18.2 MΩ ⋅ cm (at 25°C) using a Milli-Q Gradient UltraPure water purification system (Millipore, Billerica, MA) were used to prepare mobile phase solvents. Solvent A was 99.9% water–0.1% formic acid, and solvent B was 99.9% acetonitrile–0.1% formic acid (vol/vol). The elution program consisted of isocratic flow with 2% solvent B for 4 min, a linear gradient to 30% solvent B over 38 min, isocratic flow with 95% solvent B for 6 min, and isocratic flow with 2% solvent B for 12 min, at a flow rate of 300 nl/min.

Full-scan mass spectra were acquired in the positive-ion mode over the *m*/*z* range from 350 to 1,800 using the Orbitrap mass analyzer, in profile format, with a mass resolution setting of 30,000 (at *m*/*z* = 400, measured at full width at half-maximum peak height [FWHM]). In the data-dependent mode, the eight most intense ions exceeding an intensity threshold of 50,000 counts were selected from each full-scan mass spectrum for tandem mass spectrometry (MS/MS) analysis using collision-induced dissociation (CID). MS/MS spectra were acquired using the linear ion trap, in centroid format, with the following parameters: isolation width of 3 *m*/*z* units, normalized collision energy of 30%, default charge state of 3+, activation *Q* = 0.25, and activation time of 30 ms. Real-time charge state screening was enabled to exclude unassigned and 1+ charge states from MS/MS analysis. Real-time dynamic exclusion was enabled to preclude reselection of previously analyzed precursor ions, with the following parameters: repeat count of 3, repeat duration of 10 s, exclusion list size of 500, exclusion duration of 90 s, and exclusion mass width of 20 ppm. Data acquisition was controlled using Xcalibur software (version 2.0.7; Thermo). Raw data were searched against the amino acid sequence of *N. crassa* CBH-1 (NCU07340) using Proteome Discoverer software (version 1.3, SEQUEST algorithm; Thermo) for tryptic peptides with up to three missed cleavages, and carbamidomethylcysteine, methionine sulfoxide, and N-terminal pyroglutamate as variable posttranslational modifications. Peptide identifications were validated by manual inspection of the MS/MS spectra, i.e., to check for the presence of y-type and b-type fragment ions^S1^ that identify the peptide sequences. Data acquisition and integration of extracted ion chromatograms of peptide ions were performed using Xcalibur software (version 2.0.7; Thermo). MS/MS spectra are annotated using the nomenclature of Roepstorff and Fohlman (55).

### GH5-1 immunoprecipitation and Western blot analyses.

Polyclonal rabbit antibodies were obtained using the synthetic peptide DPENKIVYEMHQYLDSD from GH5-1 (Pierce Biotechnology). GH5-1-6xHis was immunoprecipitated (IP) from cell lysate using Thermo Fisher Dynabeads protein G bound to anti-GH5-1 antibody. Beads were washed and incubated for 1 h at 4°C with cell lysate, after which beads were washed with citrate-phosphate buffer (pH 5.7). Proteins were eluted with 100 mM citrate buffer (pH 2.4). Immunoprecipitated proteins were subjected to SDS-PAGE and transferred to a nitrocellulose membrane for blocking and blotting. The membrane was developed using Thermo Fisher Super Signal West Pico chemiluminescent substrate.
