# Supplementary figures and images for: Identification of Glutaminyl Cyclase Genes Involved in Pyroglutamate Modification of Fungal Lignocellulolytic Enzymes
Source: mBio. 2017 Jan 17;8(1):e02231-16. doi: 10.1128/mBio.02231-16 (PMC5241404; doi:10.1128/mBio.02231-16)

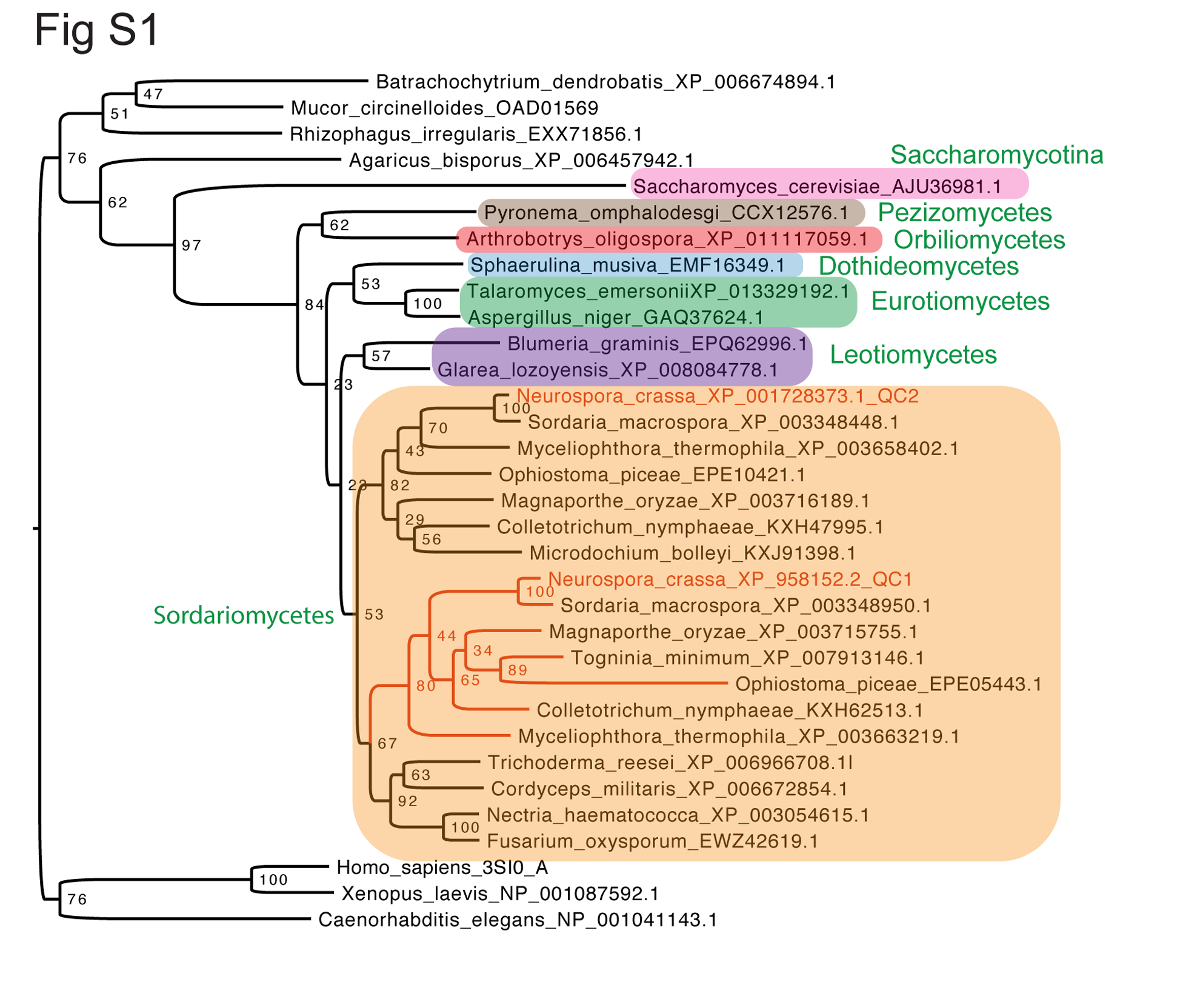

Supplement: FIG S1 [file mbo002173147sf1.tif]

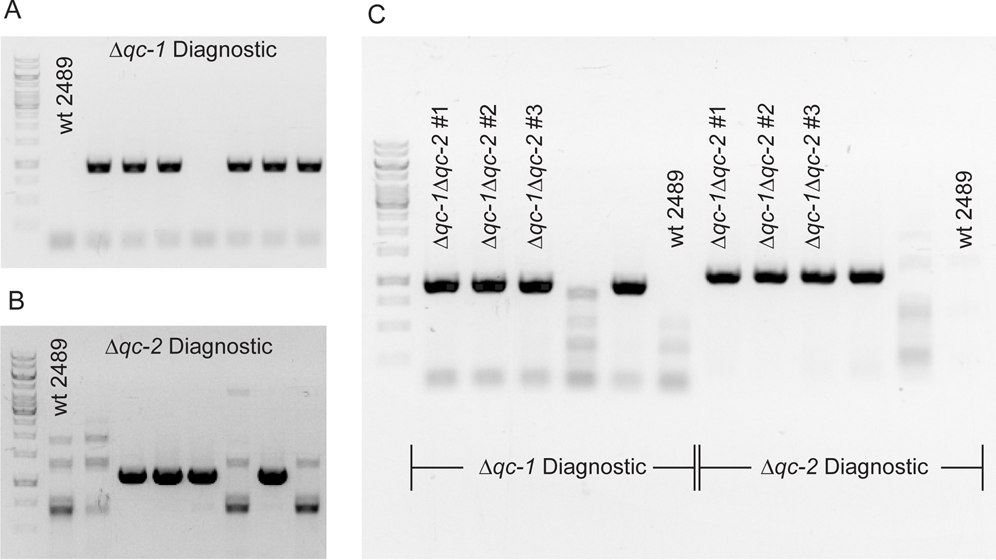

Supplement: FIG S2 [file mbo002173147sf2.tif]
