# Supplementary material for: Identification of Glutaminyl Cyclase Genes Involved in Pyroglutamate Modification of Fungal Lignocellulolytic Enzymes
Source: mBio. 2017 Jan 17;8(1):e02231-16. doi: 10.1128/mBio.02231-16 (PMC5241404; doi:10.1128/mBio.02231-16)
Supplement: TABLE S1 [file mbo002173147st1.docx]

**SI Table 1. Peptide sequences and modifications detected by LC MS/MS of trypsin digested CBH-1**

| **Trypsin digested CBH-1 purified from WT cells** | |
| --- | --- |
| **Peptide sequence^1^** | **Modifications^2^** |
| ccVDGAEYSSTYGIQTSGNSLSLQFVTK | C1(Carbamidomethyl); C2(Carbamidomethyl) |
| cTSSGcTNVAGSITVDANWR | C1(Carbamidomethyl); C6(Carbamidomethyl) |
| DSAGDLAEIK |  |
| DSAGDLAEIKR |  |
| FTVVTQFIK |  |
| FYVQNGK |  |
| GEcPTTSGVPAEVEANAPNSK | C3(Carbamidomethyl) |
| GECPTTSGVPAEVEANAPNSK |  |
| GSYSTNIGSR |  |
| INDYYSQcV | C8(Carbamidomethyl) |
| INDYYSQCV |  |
| MGNTTFYGEGK |  |
| qAVcSLTAETHPSLNWSK | N-Term(Gln->pyro-Glu); C4(Carbamidomethyl) |
| RFYVQNGK |  |
| TAFGDIDDFNK |  |
| TAFGDIDDFNKK |  |
| TVDTSSK |  |
| VIENSQSNVDGVSGNSITQSFcNAQK | C22(Carbamidomethyl) |
| VIENSQSNVDGVSGNSITQSFCNAQK |  |
| VIFSNIK |  |
| VSTAFTPHPcTTIEQHmcEGDScGGTYSDDR | C10(Carbamidomethyl); M17(Oxidation); C18(Carbamidomethyl); C23(Carbamidomethyl) |
| VSTAFTPHPcTTIEQHMcEGDScGGTYSDDR | C10(Carbamidomethyl); C18(Carbamidomethyl); C23(Carbamidomethyl) |
| VSTAFTPHPcTTIEQHMCEGDScGGTYSDDR | C10(Carbamidomethyl); C23(Carbamidomethyl) |
| WTHITSGSTNcYSGNEWDTSLcSTNTDcATK | C11(Carbamidomethyl); C22(Carbamidomethyl); C28(Carbamidomethyl) |
| WTHITSGSTNcYSGNEWDTSLcSTNTDCATK | C11(Carbamidomethyl); C22(Carbamidomethyl) |
| YGGTcDADGcDFNSYR | C5(Carbamidomethyl); C10(Carbamidomethyl) |
| YGGTCDADGcDFNSYR | C10(Carbamidomethyl) |
| YGTGYcDAQcPR | C6(Carbamidomethyl); C10(Carbamidomethyl) |
| YGTGYCDAQcPR | C10(Carbamidomethyl) |
| YINGIANVEGWTPSTNDANAGIGDHGTccSEMDIWEANK | C28(Carbamidomethyl); C29(Carbamidomethyl) |
|  |  |
| **Trypsin digested CBH-1 purified from *∆qc-1∆qc-2* cells** | |
| **Peptide sequence^1^** | **Modifications^2^** |
| ccVDGAEYSSTYGIQTSGNSLSLQFVTK | C1(Carbamidomethyl); C2(Carbamidomethyl) |
| cTSSGcTNVAGSITVDANWR | C1(Carbamidomethyl); C6(Carbamidomethyl) |
| DSAGDLAEIK |  |
| DSAGDLAEIKR |  |
| FTVVTQFIK |  |
| FYVQNGK |  |
| GEcPTTSGVPAEVEANAPNSK | C3(Carbamidomethyl) |
| GECPTTSGVPAEVEANAPNSK |  |
| GSYSTNIGSR |  |
| INDYYSQcV | C8(Carbamidomethyl) |
| INDYYSQCV |  |
| MGNTTFYGEGK |  |
| qAVcSLTAETHPSLNWSK | N-Term(Gln->pyro-Glu); C4(Carbamidomethyl) |
| QAVcSLTAETHPSLNWSK | C4(Carbamidomethyl) |
| RFYVQNGK |  |
| TAFGDIDDFNK |  |
| TAFGDIDDFNKK |  |
| TVDTSSK |  |
| VIENSQSNVDGVSGNSITQSFcNAQK | C22(Carbamidomethyl) |
| VIENSQSNVDGVSGNSITQSFCNAQK |  |
| VIFSNIK |  |
| VSTAFTPHPcTTIEQHmcEGDScGGTYSDDR | C10(Carbamidomethyl); M17(Oxidation); C18(Carbamidomethyl); C23(Carbamidomethyl) |
| VSTAFTPHPcTTIEQHMcEGDScGGTYSDDR | C10(Carbamidomethyl); C18(Carbamidomethyl); C23(Carbamidomethyl) |
| VSTAFTPHPcTTIEQHMCEGDScGGTYSDDR | C10(Carbamidomethyl); C23(Carbamidomethyl) |
| WTHITSGSTNcYSGNEWDTSLcSTNTDcATK | C11(Carbamidomethyl); C22(Carbamidomethyl); C28(Carbamidomethyl) |
| WTHITSGSTNcYSGNEWDTSLcSTNTDCATK | C11(Carbamidomethyl); C22(Carbamidomethyl) |
| YGGTcDADGcDFNSYR | C5(Carbamidomethyl); C10(Carbamidomethyl) |
| YGGTCDADGcDFNSYR | C10(Carbamidomethyl) |
| YGTGYcDAQcPR | C6(Carbamidomethyl); C10(Carbamidomethyl) |
| YGTGYCDAQcPR | C10(Carbamidomethyl) |
| YINGIANVEGWTPSTNDANAGIGDHGTccSEmDIWEANK | C28(Carbamidomethyl); C29(Carbamidomethyl); M32(Oxidation) |
| YINGIANVEGWTPSTNDANAGIGDHGTccSEMDIWEANK | C28(Carbamidomethyl); C29(Carbamidomethyl) |

^1^Sequence of each detected peptide with each modified residue in lower case.

^2^Modified residue (single letter code) followed by the position of the modified residue followed by the modification type in parentheses. Q or pGlu N-terminal peptides are highlighted in red.
