# Supplementary material for: Identification of Glutaminyl Cyclase Genes Involved in Pyroglutamate Modification of Fungal Lignocellulolytic Enzymes
Source: mBio. 2017 Jan 17;8(1):e02231-16. doi: 10.1128/mBio.02231-16 (PMC5241404; doi:10.1128/mBio.02231-16)
Supplement: TABLE S2 [file mbo002173147st2.docx]

**SI Table 2. GH5-1 peptides detected by LC MS/MS from GH5-1 digested with chymotrypsin**

| **Chymotrypsin digested GH5-1 purified from WT cells** | |
| --- | --- |
| **Peptide Sequence^1^** | **Modifications^2^** |
| AAGATSQY |  |
| AAGPWWGDYMY |  |
| AALTDPENKIVY |  |
| AcNYVNDWY | C2(Carbamidomethyl) |
| AGGANSVcKAAVTGLL | C8(Carbamidomethyl) |
| AMERLVPNTLTSSF |  |
| ANGKKGVLGEFAGGANSVcKAA | C19(Carbamidomethyl) |
| ASNSKVIFDTNNEYNTMDQTLVL |  |
| EHLKANTDVWEGALW |  |
| EMHQYLDSDSSGTSTAcVSSEIGVQRIVGATAW | C17(Carbamidomethyl) |
| ERLVPNTL |  |
| FDTNNEYNTMDQTLVLNLNQ |  |
| FGVNEAGGEFGDGIFPGRW |  |
| GKIITDTDAFKTF |  |
| GKKGVLGEF |  |
| GQcGGNGWSGATScISGY | C3(Carbamidomethyl); C14(Carbamidomethyl) |
| GTEFTFPDTNTIQTL |  |
| GVNEAGGEFGDGIFPGRWGTEF |  |
| IFVEGNQWTGAW |  |
| IITDTDAFKTF |  |
| IVLDPHNYGRY |  |
| KAAVTGLLEHL |  |
| KANTDVWEGALW |  |
| KGVLGEF |  |
| KTFWQNVAAKF |  |
| KWFGVNEAGGEF |  |
| LRANGKKGVLGEFAGGANSVc | C21(Carbamidomethyl) |
| NIFRVGF |  |
| NLNQAAIDGIRAAGATSQY |  |
| NSLLKTY |  |
| NTMDQTLVL |  |
| QAAIDGIRAAGATSQY |  |
| QNVAAKF |  |
| qSGAWGQcGGNGW | N-Term(Gln->pyro-Glu); C8(Carbamidomethyl) |
| RANGKKGVLGEF |  |
| RANGKKGVLGEFAGGANSVc | C20(Carbamidomethyl) |
| RIVGATAW |  |
| SFEPPSGTGY |  |
| SGATScISGY | C6(Carbamidomethyl) |
| TDPENKIVY |  |
| TFPDTNTIQTL |  |
| TLRSQGY |  |
| TYYNSLLKTY |  |
| VIFDTNNEYNTMDQTLVLNLNQ |  |
| VLNLNQAAIDGIRAAGATSQY |  |
| VSSEIGVQRIVGATAW |  |
| WLRANGKKGVLGEF |  |
| WQNVAAKF |  |
| YGKIITDTDAFKTF |  |
| YNSLLKTY |  |
|  | |
| **Chymotrypsin digested GH5-1 purified from *∆qc-1∆qc2* cells** | |
| **Sequence^1^** | **Modifications^2^** |
| AALTDPENKIVY |  |
| AGGANSVcKAAVT | C8(Carbamidomethyl) |
| AMERLVPN |  |
| AMERLVPNTL |  |
| ASNSKVIF |  |
| ASNSKVIFDTNNEY |  |
| ASNSKVIFDTNNEYNTM |  |
| ASNSKVIFDTNNEYNTMDQTL |  |
| ASNSKVIFDTNNEYNTMDQTLVL |  |
| EHLKANTDVW |  |
| EMHQYLDSDSSGTSTAcVSSEIGVQR | C17(Carbamidomethyl) |
| GKIITDTDAF |  |
| GKIITDTDAFKTF |  |
| GVNEAGGEFGDGIFPGRW |  |
| IFVEGNQW |  |
| IITDTDAFKTF |  |
| IVLDPHNY |  |
| KAAVTGLL |  |
| KAAVTGLLEHLKANTDVW |  |
| KANTDVW |  |
| KWFGVNEAGGEFGDGIFPGRWGTEF |  |
| LRANGKKGVLGEF |  |
| NLNQAAIDGIR |  |
| NLNQAAIDGIRAAGATSQY |  |
| NSLLKTY |  |
| NTMDQTLVL |  |
| QNVAAKF |  |
| RIVGATAW |  |
| SFEPPSGTGY |  |
| TDPENKIVY |  |
| TFPDTNTIQTL |  |
| TLRSQGY |  |
| VLNLNQAAIDGIRAAGATSQY |  |
| WQNVAAKF |  |
| YGKIITDTDAF |  |
| YGKIITDTDAFKTF |  |
| YNSLLKTY |  |

^1^Sequence of each detected peptide with each modified residue in lower case.

^2^Modified residue (single letter code) followed by the position of the modified residue followed by the modification type in parentheses. Q or pGlu N-terminal peptides are highlighted in red.
