# Supplementary material for: Identification of Glutaminyl Cyclase Genes Involved in Pyroglutamate Modification of Fungal Lignocellulolytic Enzymes
Source: mBio. 2017 Jan 17;8(1):e02231-16. doi: 10.1128/mBio.02231-16 (PMC5241404; doi:10.1128/mBio.02231-16)
Supplement: TABLE S3 [file mbo002173147st3.docx]

**SI Table 3. Predicted *N. crassa* pGlu-modified proteins.**

| **CAZy^1^** | **Broad Annotation^2^** | | **N^3^** | **FungiDB annotation^4^** |
| --- | --- | --- | --- | --- |
| NCU00206* | cellobiose dehydrogenase-1 (830 aa) | | Q | CDH-1 |
| NCU00762* | glycosylhydrolase family 5-1 (391 aa) | | Q | GH5-1 endoglucanase 2 |
| NCU02583* | glucoamylase-2 (1045 aa) | | Q | α-glucosidase |
| NCU04854* | glycosylhydrolase family 7-2 (443 aa) | | Q | GH7-2 endoglucanase 1 |
| NCU04997 | glycosylhydrolase family 10-3 (432 aa) | | Q | endo β-xylanase |
| NCU05057* | glycosylhydrolase family 7-1 (439 aa) | | Q | GH7-1 endoglucanase |
| NCU05104 | glycosylhydrolase family 7-4 (451 aa) | | Q | GH7-4 endoglucanase |
| NCU05923 | cellobiose dehydrogenase (829 aa) | | Q | CDH-2 |
| NCU06143* | hypothetical protein (1066 aa) | | Q | GH67 α-glucuronidase |
| NCU07130 | glycosylhydrolase 10-4 (446 aa) | | Q | GH10 endo β 1-4 xylanase |
| NCU07340* | cellobiohydrolase-1 (522 aa) | | Q | CBH1 |
| NCU17284 | hypothetical protein (231 aa) | | Q | β-fructofuranosidase |
| NCU00642* | glycosylhydrolase family 35-1 (1002 aa) | | E | GH35-1 β-galactosidase |
| NCU04952* | glycosyl hydrolase family 3-4 (736 aa) | | E | GH3-4 β-glucosidase |
| NCU06599 | cellulose-binding protein (269 aa) | | E | cellulose binding protein |
| NCU07811 | hypothetical protein (439 aa) | | E | cellobiose dehydrogenase |
|  |  |  | |  |
| **Fungal Cell Wall** |  |  | |  |
| NCU02579 | FAS1 domain-containing protein (229 aa) | Q | | Fas1 domain containing |
| NCU02668 | cell wall synthesis protein (446 aa) | Q | | cell wall modifier |
| NCU03318 | hypothetical protein (432 aa) | Q | | β-1,6-N-acetyl glucosaminyltransferase |
| NCU03530* | anchored cell wall protein-6 (261 aa) | Q | | chitinase 3 |
| NCU03602 | hypothetical protein (459 aa) | Q | | β-1,6-N-acetyl glucosaminyltransferase |
| NCU04160 | hypothetical protein (361 aa) | Q | | β-1,6-N-acetyl glucosaminyltransferase |
| NCU05974* | cell wall glucanosyltransferase Mwg1 (365 aa) | Q | | cell wall glucosyltransferase |
| NCU06525 | hypothetical protein (541 aa) | Q | | cell wall metabolic process |
| NCU07033 | hypothetical protein (310 aa) | Q | | ahitinase |
| NCU07269 | alpha-1,2-mannosidase (824 aa) | Q | | α 1-2 mannosidase |
| NCU07776* | anchored cell wall protein-5 (191 aa) | Q | | anchored cell wall protein |
| NCU08072 | cell wall glucanase (675 aa) | Q | | cell wall glucanase |
| NCU08127 | glycosylhydrolase family 76-3 (479 aa) | Q | | endo α 1-6 mannosidase |
| NCU09117 | extracellular cell wall glucanase Crf1 (376 aa) | Q | | cell wall glucanase |
| NCU09133* | anchored cell wall protein-7 (258 aa) | Q | | anchored cell wall protein-7 |
| NCU09171 | hypothetical protein (712 aa) | Q | | cell wall anchored protein |
| NCU09263 | anchored cell wall protein-4 (208 aa) | Q | | anchored cell wall protein-4 |
| NCU09672 | extracellular cell wall glucanase Crf1 (321 aa) | Q | | cell wall glucanase |
| NCU10416 | WSC domain-containing protein (372 aa) | Q | | WSC domain |
| NCU06185 | anchored cell wall protein-9 (246 aa) | E | | anchored cell wall 9 |
| NCU08457 | easily wettable (109 aa) | E | | fungal cell wall |
| NCU09175 | GPI-anchored cell wall beta-1,3-endoglucanase EglC (411 aa) | E | | GPI anchored cell wall endoglucanase |
| NCU05060 | hypothetical protein (469 aa) | E | | CFEM domain containing protein |
|  |  |  | |  |
| **Protease** |  |  | |  |
| NCU01351 | hypothetical protein (370 aa) | Q | | trypsin-like serine protease |
| NCU02956 | aspartic proteinase (505 aa) | Q | | aspartic proteinase |
| NCU05584 | hypothetical protein (289 aa) | Q | | aspergillopepsin |
| NCU05980 | carboxypeptidase S1 (649 aa) | Q | | serine protease-13 |
| NCU08615 | tyrosinase (701 aa) | Q | | extracellular tyrosinase |
| NCU09155 | hypothetical protein (533 aa) | Q | | aspartyl protease |
| NCU03168 | aspartic-type endopeptidase (530 aa) | E | | aspartic-type endopeptidase |
| NCU07259 | GPI-anchor transamidase (402 aa) | E | | GPI anchor transamidase |
|  |  |  | |  |
| **GPI Anchor** |  |  | |  |
| NCU01403 | GPI anchored protein (451 aa) | Q | | GPI-anchored protein |
| NCU09929 | hypothetical protein (282 aa) | E | | GPI-anchored protein |
|  |  |  | |  |
| **Oxidore-ductases** |  |  | |  |
| NCU07619 | FAD binding domain-containing protein (668 aa) | Q | | FAD binding domain containing protein |
| NCU07763 | hypothetical protein (354 aa) | Q | | NAD-dependent oxidoreductase |
| NCU09267* | copper radical oxidase (1106 aa) | Q | | copper radical oxidase |
| NCU08750 | isoamyl alcohol oxidase (515 aa) | Q | | isoamyl alcohol oxidase |
| NCU03013* | anchored cell wall protein-10 (249 aa) | Q | | Cu/Zn superoxide dismutase |
| NCU07099 | hypothetical protein (350 aa) | Q | | NAD-dependent oxidoreductase |
| NCU08173 | early conidial development-2 (406 aa) | Q | | FAD binding domain containing protein |
|  |  |  | |  |
| NCU09659 | 5'-nucleotidase (568 aa) | E | | Nucleotidase |
|  |  |  | |  |
| **ER protein** |  |  | |  |
| NCU01146 | signal sequence receptor alpha chain (273 aa) | Q | | Homolog to ER protein of unknown function |
| NCU02202 | serine/threonine protein kinase-14 (1209 aa) | Q | | IRE-1 |
|  |  |  | |  |
| **Vacuolar proteins** | |  | |  |
| NCU04090 | niemann-Pick C1 protein (1280 aa) | E | | vacuolar membrane protein |
| NCU01990 | UPF0016 domain-containing protein (506 aa) | Q | | vacuolar membrane protein |
| **OTHER** |  |  | |  |
| NCU09746 | gephyrin (726 aa) | Q | | synthesis of molybdenum cofactor |
| NCU11347 | hypothetical protein (435 aa) | Q | | transmembrane transport |
| NCU01036 | DUF907 domain-containing protein (767 aa) | E | | DUF907 domain containing |
| NCU03141 | lysophospholipase (654 aa) | E | | lypophospholipase |
|  |  |  | |  |
| **Conserved hypotheticals** | |  | |  |
| NCU01739 | hypothetical protein (207 aa) | Q | | Conserved hypothetical |
| NCU02880 | hypothetical protein (167 aa) | Q | | Conserved hypothetical |
| NCU04194* | hypothetical protein (573 aa) | Q | | Conserved hypothetical |
| NCU04603 | hypothetical protein (266 aa) | Q | | Conserved hypothetical |
| NCU05358 | hypothetical protein (350 aa) | Q | | Conserved hypothetical |
| NCU06109 | hypothetical protein (1339 aa) | Q | | Conserved hypothetical |
| NCU06801 | hypothetical protein (404 aa) | Q | | Conserved hypothetical |
| NCU07106 | hypothetical protein (260 aa) | Q | | Conserved hypothetical |
| NCU07569 | hypothetical protein (342 aa) | Q | | Conserved hypothetical |
| NCU08085 | hypothetical protein (332 aa) | Q | | Conserved hypothetical |
| NCU08438 | hypothetical protein (94 aa) | Q | | Conserved hypothetical |
| NCU08821 | hypothetical protein (917 aa) | Q | | Conserved hypothetical |
| NCU08860 | hypothetical protein (133 aa) | Q | | Conserved hypothetical |
| NCU09651 | hypothetical protein (632 aa) | Q | | Conserved hypothetical |
| NCU03512 | hypothetical protein (452 aa) | E | | Conserved hypothetical |
| NCU04373 | hypothetical protein (1074 aa) | E | | Conserved hypothetical |
| NCU05626 | hypothetical protein (391 aa) | E | | Conserved hypothetical |
| NCU05705 | hypothetical protein (129 aa) | E | | Conserved hypothetical |
| NCU06790 | hypothetical protein (282 aa) | E | | Conserved hypothetical |
| NCU09428 | hypothetical protein (403 aa) | E | | Conserved hypothetical,  possible Ca2 Transport |
|  |  |  |  |  |
| **Non-Conserved Hypotheticals** | | |  |  |
| NCU00267 | hypothetical protein (199 aa) | | Q | Hypothetical |
| NCU04752 | hypothetical protein (405 aa) | | Q | Hypothetical |
| NCU05043 | hypothetical protein (595 aa) | | Q | Hypothetical |
| NCU05229 | hypothetical protein (278 aa) | | Q | Hypothetical |
| NCU05379 | hypothetical protein (186 aa) | | Q | Hypothetical |
| NCU05395 | hypothetical protein (123 aa) | | Q | Hypothetical |
| NCU05834 | hypothetical protein (318 aa) | | Q | Hypothetical |
| NCU05917 | hypothetical protein (288 aa) | | Q | Hypothetical |
| NCU07163 | hypothetical protein (175 aa) | | Q | Hypothetical |
| NCU07475 | hypothetical protein (194 aa) | | Q | Hypothetical |
| NCU08321 | hypothetical protein (94 aa) | | Q | Hypothetical |
| NCU08521 | hypothetical protein (554 aa) | | Q | Hypothetical |
| NCU08523 | hypothetical protein (326 aa) | | Q | Hypothetical |
| NCU08646 | hypothetical protein (148 aa) | | Q | Hypothetical |
| NCU08680 | hypothetical protein (138 aa) | | Q | Hypothetical |
| NCU08681 | hypothetical protein (403 aa) | | Q | Hypothetical |
| NCU09055 | hypothetical protein (90 aa) | | Q | Hypothetical |
| NCU09448 | hypothetical protein (78 aa) | | Q | Hypothetical |
| NCU09464 | hypothetical protein (173 aa) | | Q | Hypothetical |
| NCU10027 | hypothetical protein (919 aa) | | Q | Hypothetical |
| NCU16439 | hypothetical protein (153 aa) | | Q | Hypothetical |
| NCU16443 | hypothetical protein (211 aa) | | Q | Hypothetical |
| NCU16848 | hypothetical protein (189 aa) | | Q | Hypothetical |
| NCU00757 | hypothetical protein (198 aa) | | E | Hypothetical |
| NCU02331 | hypothetical protein (156 aa) | | E | Hypothetical |
| NCU05191 | hypothetical protein (307 aa) | | E | Hypothetical |
| NCU06169 | hypothetical protein (317 aa) | | E | Hypothetical |

*^1^N. crassa* gene IDs organized by function.

^2^Gene annotations from Broad Institute genome version 12 (https://data.broadinstitute.org/annotation/genome/neurospora/MultiHome.html), some of which are outdated or mis-annotated.

^3^ Predicted glutamine (Q) or glutamate (E) resides at the N-terminus (prior to predicted pGlu formation).

^4^Annotation from FungiDB (<http://fungidb.org/fungidb/>).

*Genes with asterisks have been detected in proteomes from pervious studies.
